# Efficacy of Lactic Acid Bacteria (LAB) supplement in management of constipation among nursing home residents

**DOI:** 10.1186/1475-2891-9-5

**Published:** 2010-02-05

**Authors:** Hyang Mi An, Eun Hye Baek, Seok Jang, Do Kyung Lee, Mi Jin Kim, Jung Rae Kim, Kang Oh Lee, Jong Gi Park, Nam Joo Ha

**Affiliations:** 1 Department of Pharmacy, Sahmyook University, Seoul 139-742, Republic of Korea; 2 Department of Life Science, Sahmyook University, Seoul 139-742, Republic of Korea; 3 Eden Adventist Hospital, Gyeonggi-do 472-851, Republic of Korea

## Abstract

**Background:**

Constipation is a significant problem in the elderly, specifically nursing home and/or extended-care facility residents are reported to suffer from constipation. Lactic acid bacteria (LAB) are beneficial probiotic organisms that contribute to improved nutrition, microbial balance, and immuno-enhancement of the intestinal tract, as well as diarrhea and constipation effect. The objective of this study was to investigate the efficacy of this LAB supplement in the management of nursing home residents.

**Methods:**

Nineteen subjects (8M, 11F; mean age 77.1 ± 10.1) suffering with chronic constipation were assigned to receive LAB (3.0 × 10^11 ^CFU/g) twice (to be taken 30 minutes after breakfast and dinner) a day for 2 weeks in November 2008. Subjects draw up a questionnaire on defecation habits (frequency of defecation, amount and state of stool), and we collected fecal samples from the subjects both before entering and after ending the trial, to investigate LAB levels and inhibition of harmful enzyme activities. Results were tested with SAS and Student's t-test.

**Results:**

Analysis of questionnaire showed that there was an increase in the frequency of defecation and amount of stool excreted in defecation habit after LAB treatment, but there were no significant changes. And it also affects the intestinal environment, through significantly increase (*p *< 0.05) fecal LAB levels. In addition, tryptophanase and urease among harmful enzyme activities of intestinal microflora were significantly decreased (*p *< 0.05) after LAB treatment.

**Conclusion:**

LAB, when added to the standard treatment regimen for nursing home residents with chronic constipation, increased defecation habit such as frequency of defecation, amount and state of stool. So, it may be used as functional probiotics to improve human health by helping to prevent constipation.

## Background

Constipation is prevalent in modern societies and is a common symptom in clinical practice [1].

Constipation involves the large intestine and is a symptom rather than a disease. It is characterized by a constellation of symptoms and complaints, the most common of which are low defecation frequency (e.g. less than 3/week), irregular stool expulsion, painful and strained defecation, hard and dry stool consistency, a feeling of incomplete rectal defecation, and passing of abnormally small stools (e.g. less than 50 g/day) [2].

The prevalence of constipation and its impact on quality of life are most significant among elderly individuals, with a reported incidence among ambulatory adults 65 years of age and older of 26% in men and 34% in women. The prevalence of constipation is usually higher among elderly people living in nursing homes and hospitals than those living in the community. Once admitted, other factors may contribute to constipation (eg, changes in food and action, lack of exercise, loss of privacy or personality factors). More than 80% of nursing home and/or extended-care facility residents are reported to suffer from constipation. This population includes persons with higher frequency of risk factors (immobility, polypharmacy, and chronic medical conditions).

Dementia was reported as a risk factor for constipation, and such residents may be more difficult to manage than cognitively intact patients [3]. Other risk factors for constipation include the use of certain drugs (eg, anticholinergic antidepressants, opioid analgesics, and nonsteroidal anti-inflammatory drugs [NSAIDs] including aspirin). In clinical practice, however, the drugs that may be causing constipation may need to be continued in spite of their negative effects on bowel function. In constipation care studies, it was concluded that the nursing staffs' performance of constipation care-related tasks was time consuming and costly in the long-term care setting [4].

LAB are currently used in the prevention and treatment of disease [5,6], specifically in the intestinal environment, by inhibiting harmful bacteria through the lowering of the intestinal pH, vitamin synthesis and blood cholesterol levels. LAB are also used to treat intestinal disorders [7], for improving lactose malabsorption and immune function [8], prevention of cancer [9] and particularly to improve diarrhea or constipated conditions [7].

In the present study, we used LAB supplements containing *Lactobacillus acidophilus *(affects acute diarrhea and colitis), *Pediococcus pentosaceus *(has anti-viral effects), and *Bifidobacterium longum *SPM1205 (demonstrates in vivo inhibitory effects on harmful enzyme activities of intestinal microflora) to demonstrate any potential probiotic activity [10-12].

We aimed to investigate the efficacy of this LAB supplement in the management of nursing home residents with chronic constipation.

## Materials and methods

### Bacterial strains

The origins of the strains used in this study are shown in Table 1. For isolated of *Bifidobacteria*, fecal samples of healthy Koreans (20-30 years old) were collected by BBL's anaerobic sample collection and transport system to maintain anaerobic conditions, and were used within 24 h. Fecal samples were serially diluted 10-fold from 10^-1 ^to 10^-8^, and 100 μl was spread onto selective BL (Blood Liver) (Nissui Pharm. Co. Ltd., Japan) agar containing 5% sheep blood. After 48 hr of incubation in anaerobic conditions (90% N_2_, 5% H_2_, 5% CO_2_) (Bactron Anaerobic Chamber, Sheldon Manufacturing Inc., USA) at 37°C, brown or reddish-brown colonies 2-3 mm in diameter were selected for further identification [13].

**Table 1:** List of LAB used in this study

Bacterial strains	Source	Origin
*Lactobacillus acidophilus *(LH) CBT^a^	Commercial	NA^b^
*Pediococcus pentosaceus *(PP) CBT	Commercial	NA
*Bifidobacterium longum *SPM 1205	Isolate^c^	Human feces

^a^Purchased from CellBioTech, Kimpo, Kyunggido, Korea.

^b^Not available.

^c^Isolated from healthy Korean.

A fructose-6-phosphate phosphoketolase (F6PPK) test was performed [14] to ensure that the colonies selected were *Bifidobacteria*. To identify the isolated *Bifidobacterium *spp. at the species level, 16S rRNA sequencing was performed by Bioleaders (Daejeon, Korea).

### Participants

All the participants were recruited from the Eden Adventist Hospital in Gyeonggi-do, Korea. Male and female nursing home residents with chronic constipation, and who were presently receiving intervention (eg. laxatives, enemas and other invasive procedures, such as manual removal of fecal impaction) were evaluated for enrolment in the study. Because a significant number of the study participants were nursing home residents, it was not always feasible to obtain a diagnosis according to "Rome II" criteria for constipation as a prerequisite for the inclusion criteria. Such a diagnosis would require a level of communication with the patient that was not always possible in this study. The exclusion criteria were participants with ileus, renal failure, dialysis, Crohn's disease, ulcerative colitis and chronic abdominal pain. Thus, we screened 25 participants and included 8 male and 11 female participants that chose to participate in the study.

### Treatment

This study was designed to assess the effects of LAB (3.0 × 10^11 ^CFU/g) in the regimen of nursing home residents suffering from chronic constipation, the dispensing of standard treatment by nursing home staffs and on costs of care and medications. Participants with chronic constipation were defined as residents who used laxatives at least once a week.

The study protocol and the informed consent forms were reviewed and approved by the Eden Adventist Hospital. Each participant, or his or her legal guardian, was informed both orally and in writing. Written informed consent was obtained from all participants, or from their legal guardians, before participation in the study.

Subjects were instructed on the aim and content of the trial, as well as test methods. All 19 subjects were assigned to receive LAB twice (to be taken 30 minutes after breakfast and dinner) a day for 2 weeks in November 2008. During the trial period, the participants were prohibited from administering their existing laxative medications and/or enemas in addition to the investigational product.

### Questionnaire

Before entering the trial, the subjects completed a questionnaire on name, age, sex and current defecation habits such as frequency of defecation in four steps from 'once more than three days' to 'more than twice a day', amount of stool in three steps from 'large' to 'small', state of stool in five steps from 'like stone' to 'like water' and yes or no about 'currently taking any medicine for defecation' (Appendix 1).

Subjects were observed for any untoward symptoms such as vomiting, diarrhea or abdominal pain. The nursing home staff recorded a defecation habit diary during the study and this included information such as frequency of defecation as well as amount and state of stool passed by the subjects.

After ending the trial, the subjects draw up a questionnaire on defecation habits.

### Fecal LAB levels

We collected fecal samples from the subjects both before entering and after ending the trial, to investigate LAB levels and harmful enzyme activity.

Fecal samples (0.1 g) were suspended in 0.9 ml of 0.1 M phosphate buffer (pH 6.8 containing 0.5% cysteine) using a vortex, and 0.1 ml was then serially diluted 10-fold from 10^-1 ^to 10^-7^. 1 ml was then poured into selective MRS broth (pH 7.0) (Difco, USA). After 48 h of incubation under anaerobic conditions (90% N_2_, 5% H_2_, 5% CO_2_) (Bactron Anaerobic Chamber, Sheldon MFG. Inc., USA), colonies were counted as LAB [12]. The numbers of colony forming units (CFU) are expressed as log10 CFU per gram.

### Harmful enzyme activities of intestinal microflora

Harmful enzyme activities such as β-glucosidase, β-glucuronidase, tryptophanase, and urease of intestinal microflora related to colon cancer were tested in human fecal samples as previously described [15-17].

### Assay of β-glucosidase activity

β-glucosidase activity was assayed using 2 ml of a reaction mixture containing 0.8 ml of 2 mM p-nitrophenyl-β-D-glucopyranoside and 0.2 ml of the enzyme solution (suspended fecal sample), incubated for 30 min at 37°C, and then stopped by adding 1 ml of 0.5 N NaOH. The reaction mixture was then centrifuged at 3,000 rpm for 10 min. Enzyme activity was measured by monitoring absorbance at 405 nm.

### Assay of β-glucuronidase activity

β-glucuronidase activity was assayed using 2 ml of a reaction mixture consisting of 0.8 ml of 2 mM p-nitrophenyl-β-D-glucuronide and 0.2 ml of the enzyme solution, incubated for 30 min at 37°C, and then stopped by adding 1 ml of 0.5 N NaOH. The reaction mixture was centrifuged at 3,000 rpm for 10 min. Enzyme activity was measured by monitoring absorbance at 405 nm.

### Assay of tryptophanase activity

Tryptophanase activity was assayed using 2.5 ml of a reaction mixture consisting of 0.2 ml of complete reagent solution (2.75 mg of pyridoxal phosphate, 19.6 mg of disodium EDTA dihydrate, and 10 mg of bovine serum albumin in 100 ml of 0.05 M potassium phosphate buffer, pH 7.5), 0.2 ml of 20 mM tryptophan, and 0.1 ml of the enzyme solution, incubated for 1 h at 37°C, and then stopped by adding 2 ml of color reagent solution (14.7 g p-dimethylaminobenzaldehyde in 52 ml H_2_SO_4 _and 948 ml 95% ethanol). The reaction mixture was then centrifuged at 3,000 rpm for 10 min. Enzyme activity was measured by monitoring absorbance at 550 nm.

### Assay of urease activity

Urease activity was assayed using 0.5 ml of a reaction mixture consisting of 0.3 ml of urea substrate solution (4 mM urea in 20 mM sodium phosphate buffer, pH 7.0) and 0.1 ml of the enzyme solution, incubated for 30 min at 37°C and then stopped by adding 0.1 ml of 1 N (NH4)_2_SO_4_. Phenolnitroprusside reagent (1 ml) and alkaline hypochlorite reagent (NaClO, 1 ml) were added to the stopped reaction mixture and incubated for 20 min at 65°C. The reaction mixture was centrifuged at 3,000 rpm for 10 min. Enzyme activity was measured by monitoring absorbance at 603 nm.

### Statistical analysis

The data were collected and analyzed independently of the investigators, who did not have access to it or to its analysis. Analysis data of questionnaire were processed using the SAS, and comparisons data of fecal LAB levels and harmful enzyme activity of intestinal microflora were analyzed using the unpaired Student's t-test. Differences were considered statistically significant at *p *< 0.05.

## Results

### Analysis of questionnaire

This study tested 19 subjects with a mean age of 77.1 and suffering from chronic constipation; 8 males of mean age 77.4 and 11 females of mean age 76.9. Of these subjects, only 15 subjects (79%) were currently receiving intervention such as laxatives and/or enemas: 8 males (100%) and 7 females (64%) (Table 2).

**Table 2:** Fundamental characteristic of subjects

	Total	Male	Female
Number	19	8	11
Age	77.1 ± 10.1^a^	77.4 ± 10.7	76.9 ± 10.2
Intervention^b^	15 (79%)	8 (100%)	7 (64%)

^a^Each value provided is the mean ± standard deviation.

^b^Laxatives, enemas and other invasive procedures, such as manual removal of fecal impaction.

All 19 subjects were assigned to receive LAB (3.0 × 10^11 ^CFU/g) twice (to be taken 30 minutes after breakfast and dinner) a day for 2 weeks, and answered their defecation habits as follows; frequency of defecation: 11 individuals reported 'once more than three days' (57.9%), 3 'once two days' (15.8%), 4 'once a day' (21.1%) and 1 'more than twice a day' (5.3%) before LAB treatment; 6 'once more than three days' (31.6%), 7 'once two days' (36.8%), 6 'once a day' (31.6%) and 0 'more than twice a day' (0.0%) after LAB treatment; amount of stool: 4 individuals reported 'large' (21.1%), 11 'medium' (57.9%) and 4 'small' (21.1%) before LAB treatment; 8 'large' (42.1%), 9 'medium' (47.4%) and 2 'small' (10.5%) after LAB treatment; state of stool: 1 individual reported 'like stone' (5.3%), 5 'hard' (26.3%), 12 'soft' (63.2%), 1 'watery' (5.3%) and 0 'like water' (0.0%) before LAB treatment; 0 'like stone' (0.0%), 6 'hard' (31.6%), 11 'soft' (57.9%), 2 'watery' (10.5%) and 0 'like water' (0.0%) after LAB treatment (Table 3).

**Table 3:** Analysis of questionnaire before and after LAB treatment

		Before^a ^	After^b ^
		N = 19 (%)	N = 19 (%)
Frequency of defecation	Once more than three days	11 (57.9)	06 (31.6)
	Once two days	03 (15.8)	07 (36.8)
	Once a day	04 (21.1)	06 (31.6)
	More than twice a day	01 (05.3)	00 (00.0)

Amount of stool	Large	04 (21.1)	08 (42.1)
	Medium	11 (57.9)	09 (47.4)
	Small	04 (21.1)	02 (10.5)

State of stool	Like stone	01 (05.3)	00 (00.0)
	Hard	05 (26.3)	06 (31.6)
	Soft	12 (63.2)	11 (57.9)
	Watery	01 (05.3)	02 (10.5)
	Like water	00 (00.0)	00 (00.0)

^a^Before the experiment started.

^b^At the end of experiment.

According to the defecation habit diary recorded by the nursing home staff, frequency of defecation (*p *= 0.676), amount of stool ('large'; *p *= 1, 'medium'; *p *= 0.664 and 'small'; *p *= 0.289) and state of stool ('like stone'; *p *= 0, 'hard'; *p *= 0.109, 'soft'; *p *= 0.365, 'watery'; *p *= 0.377 and 'like water'; *p *= 0) were not statistically significant (Table 4).

**Table 4:** Analysis of defecation habit diary before, during and after LAB treatment

Variables	Total	Before^d^	During^e^	After^f^	*p *value^b^
Frequency of defecation					
Frequency	3.71 ± 2.23^a^	3.33 ± 2.50	4.07 ± 2.37	3.73 ± 1.87	NS^c^

Amount of stool					
Large	0.07 ± 0.25	0.07 ± 0.26	0.07 ± 0.26	0.07 ± 0.26	NS
Medium	2.76 ± 2.29	2.53 ± 2.72	2.53 ± 2.42	3.20 ± 1.70	NS
Small	0.89 ± 1.79	0.73 ± 1.79	1.47 ± 2.23	0.47 ± 1.13	NS

State of stool					
Hard	0.78 ± 1.26	0.47 ± 1.06	1.33 ± 1.72	0.53 ± 0.64	NS
Normal	2.40 ± 2.29	2.40 ± 2.53	1.80 ± 2.24	3.00 ± 2.07	NS
Watery	0.53 ± 1.44	0.47 ± 0.83	0.93 ± 2.28	0.20 ± 0.56	NS

^a^Each value provided is the mean ± standard deviation.

^b^Significance from each other at *p *< 0.05 as determined by Duncan's multiple-range test.

^c^NS: not significant.

^d^Before the experiment started.

^e^During the experiment period.

^f^At the end of experiment.

### Fecal LAB levels

**Figure 1:**
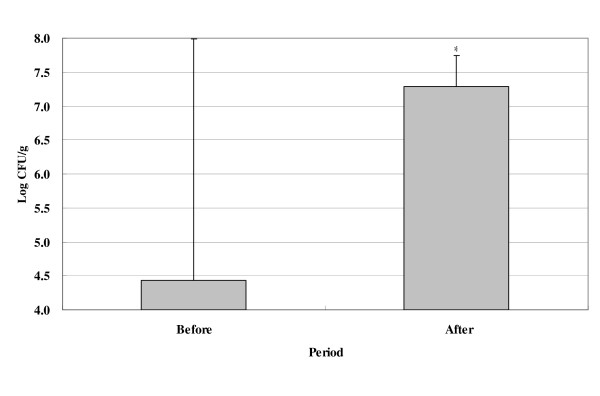
**Changes of total LAB levels in subjects**. All 19 subjects were orally administered twice (to be taken 30 minutes after breakfast and dinner) a day for 2 weeks with LAB (3.0 × 10^11 ^CFU/g). Before: before the experiment started, After: at the end of experiment. Data are presented as means and standard deviation. *p < 0.05 statistically significant compared with before LAB treatment.

Fecal LAB levels were significantly increased from 4.4 log10 CFU/g to 7.3 log10 CFU/g after LAB treatment (*p *= 0.024) (Fig. 1.).

### Harmful enzyme activities of intestinal microflora

The harmful enzyme activities of intestinal microflora were shown in Table 5. After LAB treatment, tryptophanase and urease activities were decreased by 43% and 30%, respectively. Furthermore, there was a statistically significant decrease in both tryptophanase (*p *= 0.047) and urease (*p *= 0.005) activities. However, β-glucosidase and β-glucuronidase activities were increased, and there was not a statistically significant increase in both β-glucosidase (*p *= 0.074) and β-glucuronidase (*p *= 0.061) activities.

**Table 5:** *In vivo *inhibitory effects of LAB on fecal harmful enzymes in subjects

	Period		
Activity (%)	Before^d^	After^e^	*p *value^b^
β-glucosidase	1.60 ± 1.05^a^	2.44 ± 1.05	NS^c^
β-glucuronidase	1.29 ± 0.76	2.08 ± 1.07	NS
Tryptophanase	0.41 ± 0.25	0.23 ± 0.06	0.0473
Urease	0.46 ± 0.10	0.32 ± 0.11	0.0051

^a^Each value provided is the mean ± standard deviation.

^b^Significance from each other at *p *< 0.05 as determined by Duncan's multiple-range test.

^c^NS: not significant.

^d^Before the experiment started.

^e^At the end of experiment.

## Discussion

The composition of fecal microbiota, harmful enzyme activities such as β-glucosidase, β-glucuronidase, tryptophanase, and urease of intestinal microflora, fecal frequency, and consistency were determined [18].

For this reason, we analyzed the questionnaire and tested the fecal LAB levels and harmful enzyme activities during the trials. The results showed that there were no significant changes in defecation habits, but there was an increase in the frequency of defecation and amount of stool excreted, after LAB treatment. In frequency of defecation, 'once more than three days' was decreased from 11 (57.9%) to 6 (31.6%), whereas 'once two days' and 'once a day' were increased from 3 (15.8%) to 7 (36.8%) and from 4 (21.1%) to 6 (31.6%), respectively. In amount of stool, 'small' was decreased from 4 (57.9%) to 2 (31.6%), whereas 'large' was increased from 4 (21.1%) to 8 (42.1%).

We also found that fecal LAB levels were significantly increased from 4.4 log10 CFU/g to 7.3 log10 CFU/g after LAB treatment (*p *= 0.024). That is, LAB survive passage through the upper-gastrointestinal tract after oral feeding [19], and LAB treatment affects the intestinal environment to favor LAB colonization. Ingested LAB produce lactate and SCFA (Short-Chain Fatty Acids), which can improve constipation via changes in intestinal microflora [20].

Harmful enzyme activities of intestinal microflora can implicate enterohepatic circulation of toxic and carcinogenic substances [21]. The results of the present study showed a significant decrease in the activities of tryptophanase and urease, which are harmful enzymes comprising the intestinal microflora, by 43% and 30% in subjects after LAB treatment, respectively (*p *values are 0.047 and 0.005). Thus, LAB may be potentially beneficial as functional probiotics in preventing colon cancer because of their inhibitory effects on harmful enzyme activities of intestinal microflora.

It would appear that one of the shortcomings of this study was that it was not conducted as a randomized, double-blind, placebo-controlled study. However, this was not possible given the serious health conditions and the number of subjects involved in the study.

Another possible limitation of this study was that while the difference in frequency of defecation, amount, and state of stool were evaluated, other potentially related variables such as time and sense of defecation were not evaluated. This was because of the difficulties in conversing with several subjects and the time and work-restrictions of the nursing home staff members.

LAB affected the intestinal environment by producing a clinically relevant difference in defecation habits, fecal LAB levels and harmful enzyme activities of intestinal microflora. Also, we encountered no adverse events with the daily use of LAB, which can be used alone or in combination with other previously mentioned interventions.

Thus, the results of this study showed that the LAB supplement tested, produced positive effects on the management of nursing home residents with chronic constipation.

## Conclusions

The present study demonstrated that LAB improves defecation habit (frequency of defecation, amount and state of stool) in nursing home residents with chronic constipation. In addition, this LAB improved the balance the intestinal microflora, which exert beneficial effects by decreasing harmful enzymes activities such as trytophanase and urease. Furthermore, it also affects the intestinal environment, through increase of fecal LAB levels. Therefore, LAB may be used as functional probiotics to improve human health by the management of constipation, helping to prevent colon cancer. Thus, the results of this study warrant follow-up with a larger multicenter study to further assess efficacy.

## Competing interests

The authors declare that they have no competing interests.

## Authors' contributions

This study was conceived by NJH and designed by NJH, KOL and JGP. NJH, KOL and JGP were responsible for obtaining funding and sample collection. Clinical trials, fecal LAB levels and harmful enzyme activities test were done by HMA, EHB, SJ, DKL, MJK and JRK. HMA performed data analysis and wrote the draft of the manuscript. All authors read and approved the final manuscript.

## Appendix 1 - Questionnaire

Name: ( )

Age: ( )

Sex: Male ( ), Female ( )

### * Before LAB treatment *

The following list shows the questions about currently your defecation habits.

1. Frequency of defecation?

①Once more than three days ②Once two days ③Once a day ④More than twice a day

2. Amount of stool?

①Large ②Medium ③Small

3. State of stool?

①Like stone ②Hard ③Soft ④Watery ⑤Like water

4. Are you currently take any medicine for defecate the stool?

①Yes (What is it?) ②No

### * After LAB treatment *

The following list shows the questions about your defecation habits after LAB treatment.

1. Frequency of defecation?

①Once more than three days ②Once two days ③Once a day ④More than twice a day

2. Amount of stool?

①Large ②Medium ③Small

3. State of stool?

①Like stone ②Hard ③Soft ④Watery ⑤Like water
